# Id-1 promotes migration and invasion of non-small cell lung cancer cells through activating NF-κB signaling pathway

**DOI:** 10.1186/s12929-017-0400-6

**Published:** 2017-12-12

**Authors:** Jie Li, Yingjie Li, Bin Wang, Yongfu Ma, Ping Chen

**Affiliations:** 1 Department of Chest Surgery, The General Hospital of The People’s Liberation Army, No. 28 Fuxing road, Beijing, 100853 China; 2 Department of Cardio-thoracic Surgery, First Affiliated Hospital, General Hospital of The People’s Liberation Army, Beijing, China

**Keywords:** Non-small cell lung cancer, Id-1, Migration, Invasion, NF-κB signaling pathway

## Abstract

**Background:**

Numerous studies have shown that Id-1 (Inhibitor of differentiation 1) is upregulated in several cancers and associated with tumor malignant characters. However, the clinical significance and biological role of Id-1 in non-small cell lung cancer (NSCLC) remains unclear.

**Methods:**

We used RT-PCR, Western blot and Immunohistochemistry to measure Id-1 expression in NSCLC tissues and matched adjacent noncancerous tissues. The expression pattern of Id-1 in NSCLC tissues was determined by scoring system of immunohistochemical analysis. The Kaplan-Meier method was used to calculate the survival curve, and log-rank test to determine statistical significance. The Id-1 gene was overexpressed or downreuglated with Lentiviral vectors in NSCLC cells. And, the migration ability of NSCLC cells was tested in a Transwell Boyden Chamber.

**Results:**

We found that Id-1 is generally expressed higher in NSCLC tissues compared with matched adjacent noncancerous tissues. We also found that high Id-1 expression in tumor tissues is significantly correlated with tumor progression and poor survival in NSCLC patients. Furthermore, our experimental data revealed that knockdown of Id-1 significantly suppressed the proliferation, migration and invasion of NSCLC cells, whereas ectopic expression of Id-1 promoted the malignant phenotype of NSCLC cells. Mechanistic study showed that NF-κB signaling pathway contributed to the effects of Id-1 in NSCLC cells. Moreover, blocking the NF-κB pathway significantly inhibited the tumor-promoting actions of Id-1 in NSCLC cells.

**Conclusions:**

We identified a tumorigenic role of Id-1 in NSCLC and provided a novel therapeutic target for NSCLC patients.

## Introduction

Lung cancer, predominantly non-small cell lung cancer (NSCLC) is the most commonly diagnosed malignancy, accounting for ~20% of all cancer-related death worldwide [26]. While remarkable progress during the past decades in diagnose and treatment of NSCLC, but the predicted 5-year survival rate is only around 15% [7]. And it is mainly a consequence of regional recurrence and lymph node metastasis [2, 12]. Thus, a better understanding of the molecular mechanism involved in the development and progression of NSCLC is urgently needed.

Inhibitor of differentiation 1 (Id-1), a class of dominant-negative antagonists of helix-loop-helix transcription factors, has multiple functions including inhibition of differentiation, induction of proliferation, and delaying replicative senescence [22, 29]. Recently, Id-1 has been suggested as a potential oncogene, as aberrant elevation of Id-1 has been found in numerous types of cancers such as cervical [24], osteosarcoma [9] and prostate cancers [8]. In osteosarcoma and cervical cancer patients, high Id-1 expression is correlated with more aggressive behavior as well as much shorter overall survival [9, 24]. Furthermore, Id-1 has been reported to promote survival and metastatic ability of cancer cells. For instance, Id-1 enhances invasive properties of hematopoietic cell lines through transactivation of MMP9 [21]. In addition, Id-1-induced transforming growth factor-β-induced epithelial–mesenchymal transition [14], and also enhanced cell migration in breast cancer [25]. Although Id-1 has been extensively investigated in recent years, the functions and mechanisms of Id-1 in NSCLC development remain unclear.

In the present study, we found that Id-1 overexpression is significantly correlated with tumor progression and poor survival in NSCLC patients. Gain and loss of function assays found that Id-1 promoted migration, invasion and viability of NSCLC cells by in vitro experiments. Mechanistic study showed that the tumorigenic role of Id-1 in NSCLC is at least partly via activation of NF-κB signaling pathway. Therefore, our results identify Id-1 as a novel regulator of NSCLC invasiveness and may provide a promising therapeutic strategy for treatment of NSCLC patients.

## Methods

### Patients and tumor specimens

Matched cancerous and noncancerous tissues were obtained from 96 patients with NSCLC in the Department of General surgery, The General Hospital of The People’s Liberation Army, immediately snap-frozen in liquid nitrogen, and stored at −80 °C for RNA and protein extraction. A pathologist confirmed that all the specimens were derived from normal tissues. Informed consent was obtained from the patients, and the research procedure was approved by the Ethics Committee of The General Hospital of The People’s Liberation Army.

### Cell lines and cell culture

A total of five cell lines were used including four NSCLC cell lines (H226, H292, H460 and A549) and the immortalized normal human bronchial epithelial cell line (BEAS-2B). NSCLC cells were maintained in RPMI-1640 (HyClone) supplemented with 10% FBS (Gibco) and antibiotics (100 U/ml penicillin and 100 mg/ml streptomycin) (Invitrogen). BEAS-2B cells were maintained in BEGM (Lonza). All cell lines were maintained in an incubator with a humidified atmosphere of 95% air and 5% CO_2_ at 37 °C.

### RNA extraction and quantitative real-time PCR (qRT-PCR)

The relative RNA levels of genes were assessed by quantitative real-time PCR. In brief, total RNA was isolated with the standard TRIzol-based protocol (Invitrogen, USA). RNA was reverse transcribed using the PrimeScript RT Reagent Kit (Invitrogen, USA) and qPCR was performed using SYBR Premix Ex Taq (TaKaRa, China), following the manufacturer’s instructions. The gene-specific primers were as follows: GAPDH (sense: 5’-CTGGGCTACACTGAGCACC-3′, antisense: 5’-AAGTGGTCGTTGAGGGCAATG-3′), Id-1 (sense: 5’-CCAGCACGTCATCGACTAC-3′, antisense: 5′- GCTTCAGCGACACAAGATG-3′).

### Western blot assay

Western blot analysis for specific protein expression was performed as previously described [32], while, cytoplasmic and nuclear protein were isolated and purified using the Protein and RNA Isolation System (Life technologies) according to the manufacturer’s instructions. The antibodies used were as follows: anti-Id-1 (1:500, Santa Cruz, USA), anti-p-IκBα (1:1000, CST), anti-IκBα (1:1000, CST), anti-p65 (1:1000, CST), anti- Histone 3 (1:1000, Abcam), anti-E-cadherin (1:1000, CST), anti-N-cadherin (1:1000, CST) and anti-β-Actin (1:1000, Santa Cruz, USA). The signals were detected by enhanced chemiluminescence (Pierce, USA).

### Immunohistochemistry (IHC) staining

The NSCLC and normal lung tissues were fixed, embedded, sectioned, and deparaffinized. Some of the deparaffinized sections were stained with H&E staining. IHC staining was performed using a Dako Envision System (Dako, USA) following the manufacturer’s protocol. Sections were blocked using serum-free protein block buffer (DAKO, CA, USA) for 30 min, after which they were incubated with anti-Id-1 (1:200, Santa Cruz, USA).

### Stable cell lines construction

Full-length human Id-1 cDNA was compounded by Genechem and ligated into pENTR221 vector (Genechem, Shanghai). An empty vector was used as the negative control. The Id-1 knockdown was achieved by RNA interference using a lentiviral vector-based shRNA (Genechem, Shanghai). Lentiviral particles corresponding to the shRNA Id-1 NM_002165 target set were used, as well as a nontarget shRNA control. The shRNA sequences were as follows: Id-1 shRNA, CGTGCTCTGTGGGTCTC. For the generation of stable cell lines, Lv-Id-1 or sh-Id-1 was transfected into the NSCLC cells, and selected with puromycin. Specificity and efficacy of the lentivirus were evaluated by Western blotting after transduction and puromycin selection in cells.

### Cell proliferation assays

To examine cell proliferation, 5 × 10^3^ cells/well were seeded in a 96-well plate and cultured in a 5% CO_2_ incubator at 37 °C. At the indicated time points, viable cells were counted using the Cell Counting Kit-8 according the manufacturer’s protocol.

### Colony formation assay

Cells were seeded in 6-well plates at a density of 1000 cells per well. After incubation for 14 days, the supernatant was discarded and cells were washed twice in phosphate-buffered saline (PBS). Then, cells were fixed in 10% methanol for 15 min and stained with Giemsa for 20 min. Colonies consisting of more than 50 cells were scored, and the rate of colony formation was calculated.

### Cell migration and invasion assay

The migration ability of NSCLC cells was tested in a Transwell Boyden Chamber (8-mm pore size, BD Biosciences) as previously described [10]. For the cell invasion assay, the polycarbonate membranes of the upper compartment of the chambers were precoated with a matrix gel.

### Immunofluorescent analysis

The Cells (5× 10^3^) were implanted onto a slide for 24 h, and then were fixed with paraformaldehyde for 30 min, followed permeabilizing with 0.3% Triton X-100/PBS for 5 min at room temperature. Then, cells were blocked with 5% BSA for 1 h at room temperature and stained with anti-E-cadherin (1:200, CST) and anti-N-cadherin (1:200, CST) at 4 °C overnight, followed by incubation with fluorescent-dye conjugated secondary antibody (1:200, Invitrogen) for 1 h, and then stained with DAPI. Fluorescence images were photographed with a confocal microscope.

### Statistical analysis

All data are expressed in terms of means ± the SEM. Significant differences were analyzed using Student’s t-test and two-tailed distribution. The Kaplan-Meier method was used to calculate the survival curve, and log-rank test to determine statistical significance. *P* values < 0.05 were considered statistically significant.

## Results

### Id-1 is upregulated in tumor tissues and closely correlated with clinical outcomes of patients with NSCLC

**Fig. 1:**
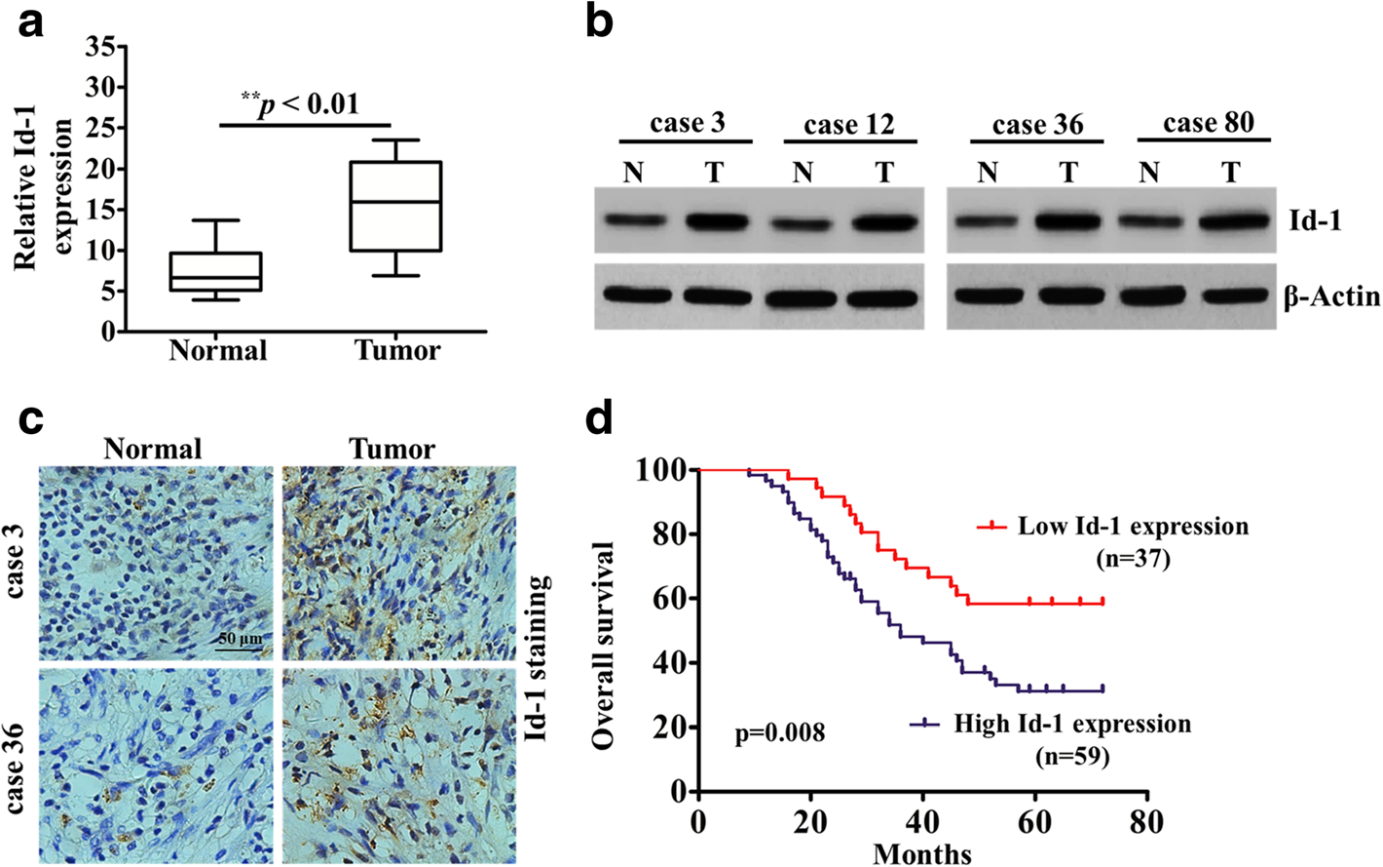
Relative Id-1 expression in NSCLC clinical samples, and its clinical significance. **a** Relative mRNA levels of Id-1 in NSCLC tissues and in paired noncancerous tissues. Id-1 expression was examined by qPCR and normalized to GAPDH expression. ^**^ *P <* 0.01, compared with non-tumor counterpart. **b** Western blot of Id-1 protein quantification in 4 NSCLC tissues and in paired noncancerous tissues. β-Actin was a loading control. **c** Representative images of Id-1 staining in 96 paired NSCLC tissues. Scale bar, 50 μm. **d** Kaplan-Meier curves for overall survival of two groups defined by high and low expression of Id-1 in patients with NSCLC. ^**^ *p* = 0.008

To investigate the potential role of Id-1 in NSCLC development, we firstly measured the expression of Id-1 in paired tumor tissues and matched adjacent noncancerous tissues from 96 patients with NSCLC using qRT-PCR. As shown in Fig. 1a, the expression of Id-1 was significantly upregulated in tumor tissues compared with the adjacent noncancerous tissues in these 96 NSCLC patients. Furthermore, we randomly selected four tissue samples of NSCLC and paired normal lung according to the results of qRT-PCR analysis to analyze the expression of Id-1 protein. Consistently, the results showed that the expression of Id-1 protein was also enhanced in NSCLC tissues in comparison with the adjacent noncancerous tissues by western blot assay (Fig. 1b). Moreover, these findings were confirmed by detecting Id-1 protein expression by immunohistochemical (IHC) staining. As shown in Fig. 1c, the data revealed that Id-1 was overexpressed in 61.5% (59/96) NSCLC specimens detected.

Next, we analyzed associations between Id-1 expression and clinicopathological parameters in NSCLC patients detected (Table 1). The results showed that the upregulation of Id-1 were significantly associated with larger tumor size (*p* = 0.028), lymph node metastasis (*p* = 0.018) and advanced TNM stage (*p* = 0.005). In addition, the Kaplan-Meier survival curves (Fig. 1d) revealed that high level of Id-1 expression had a shorter survival time than patients who had low level of Id-1 expression (*n* = 96, *p* = 0.008). Taken together, these findings suggest that the expression of Id-1 was significantly upregulated in NSCLC tissues and implicated in the progression of NSCLC.

**Table 1:** The relationship between Id-1 expression and clinicopathological factors in 96 NSCLC patients

Pathological characteristics	No. 96	Id-1 expression		*P* value
		High, n	Low, n	
Age, years				
≤ 55	45	30	15	
> 55	51	29	22	0.325
Gender				
Male	57	39	18	
Female	39	20	19	0.090
Tumor size(cm)				
≤ 3.5	41	20	21	
> 3.5	55	39	16	**0.028** ^*^
TNM stage				
I-II	46	35	11	
III-IV	50	24	26	**0.005** ^**^
Smoking history				
No	38	26	12	
Yes	58	33	25	0.257
Lymph node metastasis				
Negative	40	19	21	
Positive	56	40	16	**0.018** ^*^
Histopathologic type				
Adenocarcinoma	41	23	18	
Non-adenocarcinoma	55	36	19	0.351

^*^ ***P***<0.05 or ^**^ ***P***<0.01, statistically significant

### Id-1 promotes cell viability, migration and invasion of NSCLC cells

**Fig. 2:**
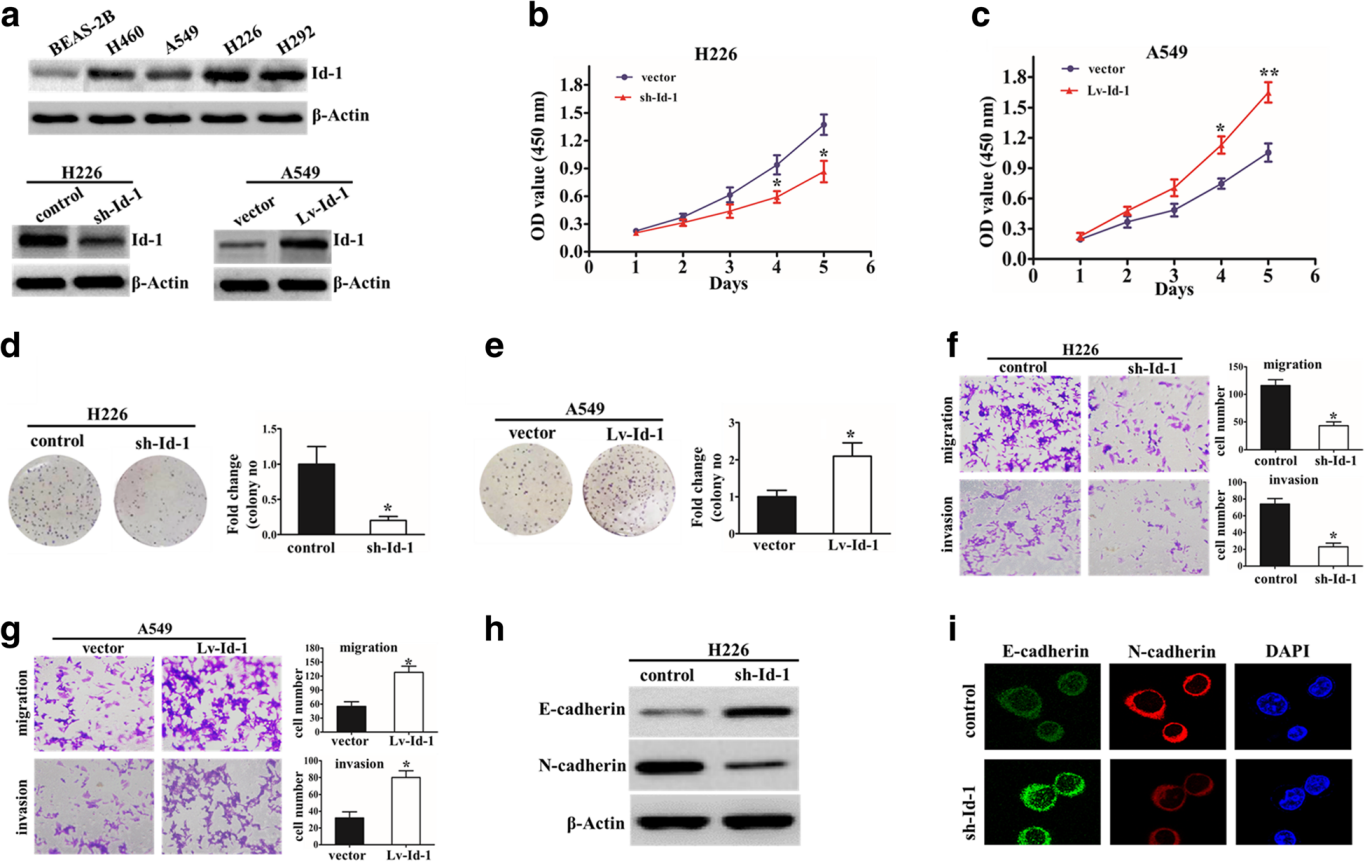
Id-1 was associated with viability and mobility features of NSCLC cell. **a** Determination of Id-1 expression levels in four NSCLC cell lines and the immortalized normal human bronchial epithelial cell line (BEAS-2B). The efficiency of Id-1 silencing and overexpression in NSCLC cell lines was measured by Western blot. β-Actin was a loading control. **b** and **c** Representative results for cell proliferation rate were evaluated in Id-1-knockdown (**b**) or Id-1-overexpressing (**c**) NSCLC cells by using CCK-8 assay. ^*^ *p* < 0.05, ^**^ *p* < 0.01. **d** and **e**, Representative images (left) and quantification (right) of the clone formation assays are shown in Id-1-knockdown (**d**) or Id-1-overexpressing (**e**) NSCLC cells. ^*^ *p* < 0.05, compared with control groups. F and G, Representative results (right) and Quantification (left) of the migration and invasion showing the effect of Id-1 knockdown (**f**) or Id-1 overexpression (**g**) on the migratory abilities of NSCLC cells. ^*^ *p* < 0.05. **h** Western blot analysis of the phenotypic markers, including E-cadherin and N-cadherin in Id-1 silencing cells. β-Actin was used as the loading control. **i** Confocal microscopy analysis of the E-cadherin and N-cadherin. The red and green signal represents the staining of the corresponding protein, and the blue signal represents the nuclear DNA staining by DAPI

To further explore the biological function of Id-1 in NSCLC, we initially measured the expression level of Id-1 in four NSCLC cell lines (A549, H460, H292 and H226) and human bronchial epithelial cell line (BEAS-2B). As shown in Fig. 2a, the expression of Id-1 was significantly higher in four NSCLC cells than compared with BEAS-2B cell. Interestingly, the expression of Id-1 was much higher in NSCLC cell lines derived from metastatic sites than that derived from primary sites (Fig. 2a). Then, we knocked down Id-1 by stably expressing Id-1 shRNA in H226 cells, which normally show relatively high Id-1 expression (Fig. 2a). Meanwhile, we developed stable clones with Id-1 overexpression from A549 cell, which exhibit relatively low expression of Id-1 among NSCLC cell lines (Fig. 2a).

Next, we examined the effects of Id-1 on the NSCLC cell’s viability and metastasis. Our results show that H226/sh-Id-1 cells showed a significantly lower in vitro proliferation rate than control cells (Fig. 2b). Conversely, the overexpression of Id-1 significantly increased cell viability of A549 cell (Fig. 2c). Meanwhile, knockdown of Id-1 resulted in a substantial decrease in H226 cell growth in comparison with control groups, whereas overexpression of Id-1 promotes the proliferation of A549 cells by colony formation assay (Fig. 2d and e).

In addition, we investigated the effect of Id-1 on migration and invasion of NSCLC cells. The results demonstrated that knockdown of NSCLC significantly suppressed the cell migration and invasion capability of H226 cells (Fig. 2f), whereas overexpression of Id-1 promoted migration and invasion ability of A549 cells (Fig. 2g). It is well known that EMT is the remarkable presentation for cell metastasis, whether Id-1 knockdown reverse the EMT phenotype need to be identified. As showed in Fig. 2h, western blot revealed that N-cadherin was downregulated after Id-1 knockdown, while E-cadherin was increased in H226 cells. Immunofluorescence assays also confirmed that down-regulation of Id-1 increased the epithelial marker but decreased the mesenchymal maker in H226 cells (Fig. 2i). Therefore, these results demonstrated that down-regulation of Id-1 inhibited NSCLC cell migration and invasion by suppressing the EMT.

### Id-1 enhances IκBα phosphorylation and NF-κB activation in NSCLC cells

**Fig. 3:**
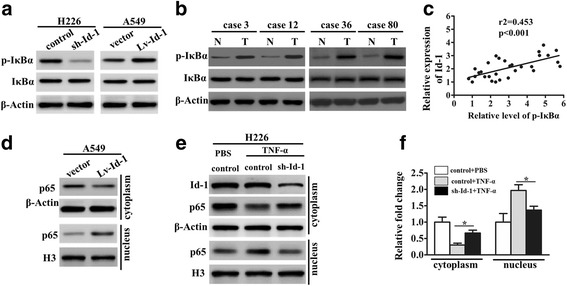
Id-1 positively regulate IκB phosphorylation and NF-κB activation in NSCLC cells. **a**, Western blotting showing total and phosphorylated IκBα in Id-1-silenced H226 cells and Id-1-overexpressing A549 cells. β-Actin was a loading control. **b**, Western blotting showing total and phosphorylated IκBα in NSCLC tissues. β-Actin was a loading control. **c**, Scatter plots showing the positive linear correlation between the Id-1 expression and phosphorylation level of IκBα in NSCLC tissues. **d**, Western blot for nuclear and cytoplasm p65 in Id-1-overexpressing A549 cells. β-Actin and Histone 3 (H3) is the loading control for cytoplasm and nuclear, respectively. **e**, Western blot for Id-1, nuclear and cytoplasm p65 in Id-1- silenced H226 cells after incubation with TNF-α (10 ng/ml) for 24 h. β-Actin and Histone 3 (H3) is the loading control for cytoplasm and nuclear, respectively. **f**, Graphic presentation of the relative abundances (fold induction versus the controls).^*^ *P* < 0.05 versus control groups after TNF-α treatment

To elucidate the underlying mechanism of Id-1-mediated tumorigenesis of NSCLC, we examined the activation of multiple signaling pathways. Among the pathways we screened, the NF-κB signaling pathway was found to be involved in Id-1-mediated function. We found that the phosphorylation level of IκBα was significantly reduced in Id-1-knockdown cells, whereas it was elevated in Id-1-overexpressing cells (Fig. 3a). Furthermore, we measured the phosphorylation level of IκBα (activation of NF-κB signaling pathway) in NSCLC tissues, which exhibited high Id-1 protein expression. Our results indicate that the elevated phosphorylation level of IκBα was accompanied with high Id-1 expression in NSCLC tissues, when compared with corresponding non-tumor tissues (Fig. 3b). Moreover, Id-1 expression was positively correlated with phosphorylation level of IκBα in NSCLC tissues (Fig. 3c).

Next, we detected the effect of Id-1 on the activation of NF-κB in NSCLC cells. As shown in Fig. 3d, overexpression of Id-1 significantly improved the nuclear translocation of p65. Moreover, TNF-α (10 ng/ml) was used to induce the NF-κB activation in Id-1-knockdown cells. The results showed that knockdown of Id-1 led to significant cytoplasm retention of p65 after TNF-α treatment compared with control groups (Fig. 3e and f). Therefore, these findings suggest that Id-1 promoted phosphorylation of IκBα and p65 nuclear translocation in NSCLC cells.

### Id-1 promotes migration and invasion of NSCLC cells through NF-κB signaling pathway

**Fig. 4:**
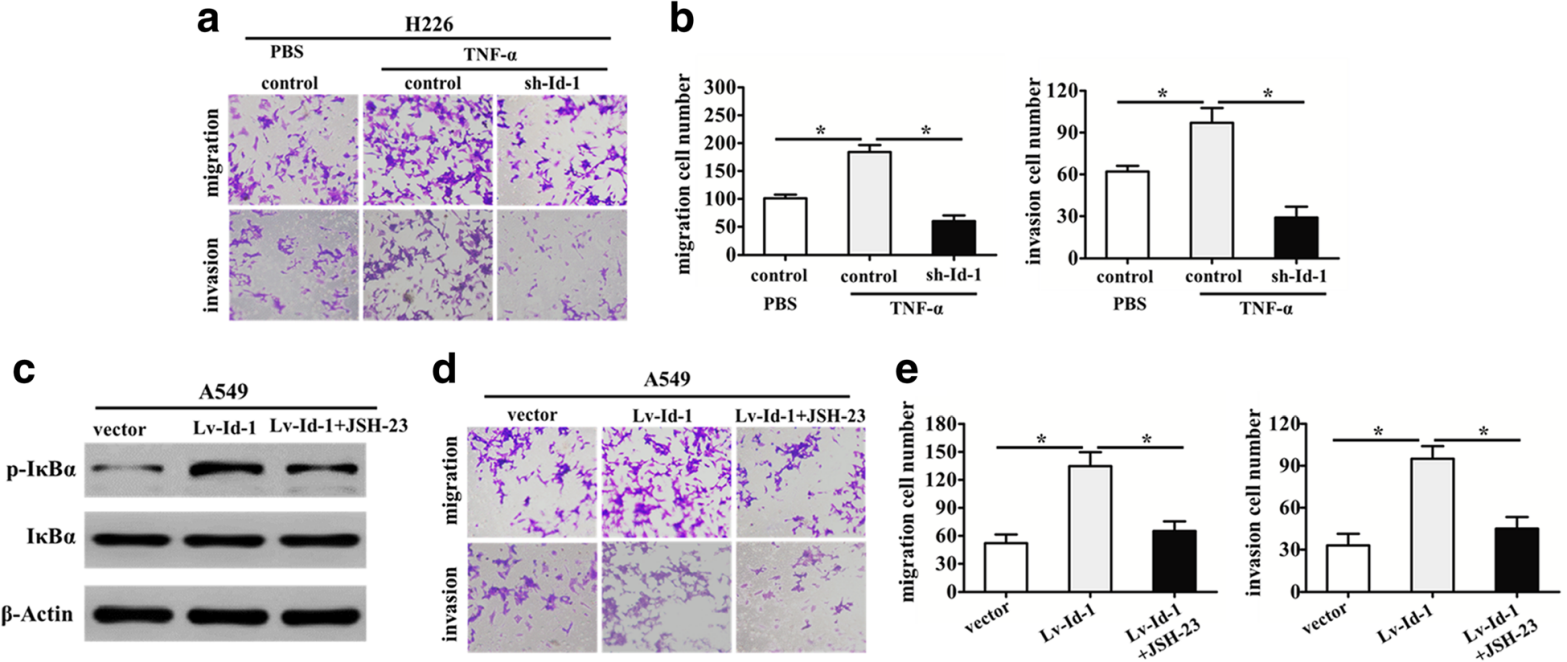
The NF-κB signaling pathway is involved in Id-1-mediated invasiveness of NSCLC cells. **a** and **b** Representative results (**a**) and Quantification (**b**) of migration and invasion of H226 cells, which were stably transfected with sh-Id-1 or negative control with or without TNF-α stimuli measured by Chamber assay. ^*^ *p* < 0.05. **c** Western blotting showing total and phosphorylated IκBα in Id-1-overexpressing A549 cells after treatment without or with JSH-23 (JSH). β-Actin was a loading control. **d** and **e** Representative results (**d**) and Quantification (**e**) of migration and invasion of A549 cells stably expressing Id-1 with or without JSH-23 measured by Chamber assay. ^*^ *p* < 0.05

To further evaluate whether NF-κB signaling pathway is responsible for NSCLC cell migration and invasion regulated by Id-1, we performed rescue experiments by treatment with NF-κB activator TNF-α and NF-κB translocation inhibitor JSH-23. As shown in Fig. 4a and b, the increased cell migration and invasion ability induced by TNF-α in H226 cells was partly abolished by Id-1 knockdown. Then, we examined the phosphorylation level of IκBα (activation of NF-κB signaling pathway) in present of inhibitor JSH-23. As shown in Fig. 4c, the elevated phosphorylation level of IκBα was abolished in A549 cells after treatment with inhibitor JSH-23. As expected, the upregulation of Id-1 significantly improved the migration and invasion capacity of A549 cells, which was significantly abrogated by NF-κB translocation inhibitor JSH-23 (Fig. 4d and e). Collectively, all these results suggested that NF-κB pathway acts as the downstream component of Id-1 and contributes to the effects of Id-1 in NSCLC cells.

## Discussion

Id-1 has been reported to be upregulated and identified as a potential tumor promoter in diverse cancers, including hepatocellular carcinoma and breast cancer [6, 31]. However, it is worth noting that the underlying biological role of Id-1 in NSCLC is still unknown. Here, our findings showed that Id-1 expression remarkable increase in NSCLC tissues compared to the corresponding non-tumor tissues. Importantly, we revealed that high expression level of Id-1 is closely associated with tumor size, high stage, lymph node metastasis, and as an independent risk factor to predict overall survival. These data indicated that high Id-1 expression may represent a novel indicator of poor prognosis in NSCLC and may function as an oncogene in NSCLC progression.

Increasing evidence has demonstrated that Id-1 is involved in the regulation of cancer cell proliferation. Hao et al. have reported that Id-1 promotes osteosarcoma cell growth and inhibits cell apoptosis via PI3K/AKT signaling pathway [9]. Additionally, Id-1 induces ESCC cell proliferation may be through inactivation of p16^INK4A^/RB pathway [13]. In present study, our experimental data revealed that overexpression of Id-1 significantly enhanced cell proliferation by activation of NF-κB signaling pathway in NSCLC cells. Based on these results, we suggest that the downstream signaling pathways of Id-1-mediated cell proliferation may differ between cancer types.

It is known that metastasis is one of the major characteristics in many cancers, and is associated with cancer progression [27, 30]. Previous studies have demonstrated that Id-1 is suggested to play a critical role during invasion and metastasis in various cancers [11, 18]. For example, Id-1 silencing significantly suppressed the gastric cancer cell migration and invasion in vitro [18]. Cao Y. et al. have reported that fucoidan-induced Id-1 suppression inhibited the in vitro and in vivo invasion of hepatocellular carcinoma cells [4]. Consistent with these studies, our experimental data revealed that downregulation of Id-1 also inhibited migratory ability of NSCLC cells. Thus, these findings suggest that Id-1 would be a potential anti-metastasis therapeutic target for NSCLC patients.

Since Id-1 is a regulator of transcription, it may be responsible for regulation of gene expression involving multiple transduction pathways. So far, several signaling pathways have been suggested to mediate the tumorigenic effects of Id-1 [15, 23]. Id-1 has been shown to promote cell survival by activating NF-κB signaling pathway and it has been suggested that it may be one of the upstream regulators of NF-κB in various cancer [16, 17]. Consistent with this findings, we also found that the NF-κB signaling pathway is responsible for Id-1-mediated tumor cell migration and invasion. As a transcription factors, NF-κB regulates many downstream genes expression including that regulate cell metastasis and plays pivotal roles in both promoting and maintaining an invasive phenotype [3, 20]. Especially, a lots of evidence showed that NF-κB signaling pathway activates the expression of genes important for the progression of NSCLC [5]. For instance, Foxp3 downregulation in NSCLC mediates epithelial-mesenchymal transition via NF-κB signaling pathway [28]. The major finding of this study is that the oncogenic effect of Id-1 is through activation of NF-κB signaling pathway leading to promotion of metastasis in NSCLC cells. On the basis of these findings, Id-1 could be a promising new therapeutic target for suppression the metastasis of NSCLC. Additionally, a previous study agreed with these conclusions revealing that Id-1 is able to protect anticancer drug induced apoptosis through activation of NF-κB pathways in prostate cancer [17]. The NF-κB inhibitor JSH-23 has been evaluated for antitumor activity in a variety of preclinical cancer models [1, 19], but has not been extended to NSCLC. Here, we found that blocking NF-κB signaling pathway using JSH-23 attenuated the Id-1-induced tumor cell metastasis, provide novel evidence to support further explorations into the use of NF-κB inhibitors in NSCLC cancer therapy. Taken together, the tumor-promoting effects of Id-1 in NSCLC cells is at least partly through activation of NF-κB signaling pathway.

## Conclusions

In summary, our data revealed that Id-1 was upregulated in NSCLC tissues and cell lines. We also exhibited that Id-1 may be a potential inducement in NSCLC invasiveness though activation of NF-κB signaling pathway. On the basis of these findings, Id-1 could be a promising new therapeutic target for suppression the metastasis of NSCLC.
